# Reducing greenhouse gas emissions in a bariatric surgical unit is a complex but feasible project

**DOI:** 10.1038/s41598-024-51441-9

**Published:** 2024-01-13

**Authors:** Jerome Dargent

**Affiliations:** Polyclinique de Rillieux, 65 Rue des Contamines, 69140 Rillieux-la-Pape, France

**Keywords:** Obesity, Climate-change mitigation

## Abstract

Obesity is a growing issue worldwide, whose causes and consequences are linked to the environment and which therefore has a high carbon footprint. On the other hand, obesity surgery, along with other procedures in surgical suites, entails environmental consequences and responsibilities. We conducted a prospective comparative study on two groups of bariatric interventions (N = 59 and 56, respectively) during two consecutive periods of time (Oct 2021–March 2022), first without and then with specific measures aimed at reducing greenhouse gas emissions related to bariatric procedures by approximately 18%. These measures included recycling of disposable surgical equipment, minimizing its use, and curbing anesthetic gas emissions. Further and continuous efforts/incentives are warranted, including reframing the surgical strategies. Instead of comparing measurements, which is difficult at the present time, we suggest defining an ECO-SCORE in operating rooms, among other healthcare facilities.

## Introduction

There is no need to dwell on the global obesity epidemic in 2023 and its many connections with environmental issues. It has been estimated that the economic impact of overweight and obesity will grow from 2.19 to 3.3% of gross domestic product in > 160 countries by 2060^1^. While the development of obesity inherently involves a waste of natural resources, the health consequences of obesity also have severe repercussions, and bariatric surgical procedures, in their current configuration, undoubtedly have their share of responsibility. Indeed, obesity surgery itself generates a significant proportion of global greenhouse gas emissions (GHGe) to which operating rooms (OR) have been shown to contribute greatly^2–4^. The time has now come for bariatric specialists to take an in-depth look at the relationship between these issues and how they should be addressed.

## Operating room and environment

Depending on calculations, the healthcare sector is responsible of up to 10% of total GHGe (USA, UK)^2,3^, which should raise awareness and call for urgent action^4^. It has been shown that ORs were a major source of GHGe worldwide, representing up to 60% of the emissions of a given hospital^3^; their carbon footprint has been estimated at approximately 184 kg per intervention, which corresponds to the weekly consumption of a 4-person family in the western world^4^. Depending on the operations, locations, and calculation methods, this footprint varies from 6 to 814 kg^3^. Among others, surgical operations account for 21–30% of hospital waste, and electricity alone represents more than 60% of the total^2,3^. There are three different scopes of GHGe in ORs (Scope 1: anesthetic gases; Scope 2: electricity use and heating; Scope 3: surgical supply chain and waste disposal), and it has been recommended to act separately on each of them by MacNeill et al.^2^.

According to a meta-analysis by Rizan et al.^3^, the carbon footprint of surgery can be reduced by improving the energy efficiency of ORs, using reusable or reprocessed surgical devices, and streamlining common procedures. While multiple approaches need to be combined, some limits have been encountered: commonly implemented means, such as recycling surgical waste, can result in a reduction in GHGe of less than 5%^5^.

Acting upon the elements that contribute to this situation proves difficult because of the great heterogeneity of potential measures that can be implemented in various locations^2^. In a study by Thiel, the total carbon footprint generated by a laparoscopic hysterectomy was estimated at 562 kg and could be reduced to 285 kg and even to 125 kg if anesthesia was removed from the equation^5^. Anesthetic gas actually represents a very significant part of these emissions, as illustrated for example in 2020 by Ryan et al., who emphasized the global warming potential of sevoflurane^6^.

Relevant leads have been suggested in order to decrease GHGe^5^: (1) minimizing the materials used in the OR, (2) maximizing instrument reuse and/or single-use instrument reprocessing, (3) moving away from some heat-trapping anesthetic gases, (4) reducing off-hour energy use in the OR. As a major user of those facilities and materials, bariatric surgery is a good example of the potential for savings in this regard.

Hence, we should answer the following questions:

- Can we clear a path towards less GHGe in a regular operating room, on a larger scale than currently and in a timely manner?
- Can we elaborate a consensus regarding the best available options, despite the complexity of existing guide-lines, various focuses and regulations?
- Do current habits take a reasonable path in terms of recycling, sparing resources, and finally looking for less energy-consuming procedures?
- How do the specificities of bariatric/metabolic procedures impact this reasoning?
- Ultimately, can we reduce the GHGe in the theatre by more than 10% for the time being?

## Material and methods

Following the steps described in previous studies^2–5^ and taking into account local possibilities, we have identified several measures that could be implemented in our center and its bariatric component, with a combination of approaches: (1) original measures pertaining to recycling and maximizing instrument use; (2) improvements in waste management that could have been implemented earlier, as other centers have done; (3) relevant measures that should be implemented in the near future but have not been so far because of more or less temporary conditions at the local level or due to the current health regulations; (4) decreased use of anesthetic gas through new ways. The methods are listed in Table 1.

**Table 1:** Calculations.

Calculation methods: General^7^	
1 kwH electricity = 0.0217 kg/ plastic production, 1 t = 3116 kg, metal production 1 t = 12,870 (3–12), paper and plaster 1 t = 872 kg	
Calculation methods: 1. Waste and recycling	
Waste for a typical intervention, i.e. laparoscopic sleeve gastrectomy (yellow bin, contaminated): 1.45 kg (surgical) + 0.7 kg (anesthesia) + 1.2 kg (medical devices, container) = 3.3 kg = 0.3 kg CO_2_ eq/0.75 kg (black bin) + 0.4 kg (anesthesia) = 1 kg = 0.02 kg CO_2_ eq. Material provided for a sleeve gastrectomy is rather similar to gastric bypass (staplers and energy), whereas it is much less for a band removal (−60%). Given the important difference in GHGe between the two kinds of waste, we considered a more stringent way of separating them, according to recent guide-lines that were in favor of diminishing the contaminated part	
1 t waste = 21.35 kg CO_2_ (10 kg = 0.2), yellow bin × 5	
Medical device recycling: 1 t metal = 4302 kg CO_2_ eq. (construction), 1 t plastic = 3116 kg CO_2_ eq. 1 laparoscopic sleeve gastrectomy = 4.3 kg = 14 kg CO_2_ eq	
Calculation methods: 2. Minimizing instrument use	
Autoclave = a cycle lasts 80 min and consumes 20 kWh. The spared volume can be estimated at 6% of the load for one cycle. 20 kwH for 1 cycle. Energy spared if 1 small container is used instead of 1 mid-size container = 0.5/7.5 = 6% = 1.2 kwH	
Method: the essential instruments for a given intervention were preserved, additional instruments were available in or out of the operating room upon request. No waste of time results from this strategy, except reassessing the instrument-boxes beforehand	
Calculation methods: 3. Anesthetic gas recycling	
In most theatres, virtually all inhaled anesthetic agents are released in the atmosphere, because no mandatory capture device is inserted into the anesthetic respiratory machine. The anaesthetic gas scavenging system (AGSS) represents an improvement and has been conceived to protect the OR personnel, but still releases gas outside and causes energy expenses due its motorization	
The CONTRAfluran™ anesthetic gas capture system (Baxter company) collects exhaled desflurane and sevoflurane in the surgical suite^8^. It is made of a canister that contains a porous material absorbing and retaining anesthetic gas, and is attached directly to the breathing machine. Once the canister is full (< 240 cc), it is collected and recycled for reuse. It can be used as an alternative or as an adjunct to the AGSS	
It has been tested in a nearby hospital belonging to same group of private hospitals as ours, and for various administrative reasons, it could not be used in ours during the time of the study. Ad hoc measurements could be done, thereby simulating actual results in our facilities. 1 canister was retrieved for 2 reloads of sevoflurane, i.e. 44 kg CO_2_ eq. 1 reload = approx. 10 h intervention. If the AGSS is stopped, + 22 kg CO_2_ spared for the same use	
Calculation methods: 4. Others	
Given the current situation of electricity supply and the important variations in the energy mix, calculations may be irrelevant within the framework of this study. Moreover, current recommendations regarding energy savings (e.g. limited night-time air-conditioning, off-hour heating, ventilation, etc.) were already in effect at the time of the study	
Efforts concerning laundry and surgical gowns/drapes have been approximated at the time of their initiation	

Some measurements have been suggested based on available data, i.e. the DD UK tables^7^. In this regard, it appeared that calculations concerning expensive and disposable instruments that are widely used in bariatrics (energy devices, staplers) were most meaningful, while others remained elusive.

## Building a surgical ECO-SCORE

According to some reports for the industry in general, up to 60% of saved emissions may be obtained through inter-industrial cooperation, including 35% of recycling and of 5% energy consumption. These figures are actually difficult to extrapolate to the health sector, which displays great differences from one hospital or group of hospitals to another, as exemplified in Mac Neil’s paper^2^.

Notwithstanding, hospitals could share mutual standards in many respects and despite their differences, from anesthetic gas to instrument reusing/reprocessing, with specific goals. Although some hospitals or groups of hospitals issued environmental or “responsibility” claims, it is difficult to assess whether or not a real green strategy has been implemented.

We suggest an ECO-SCORE including several indicators that would demonstrate a favorable trend starting from a given setting and would encompass the current variations among hospitals, areas, countries, etc. It seems reasonable to address several levels of objectives according to the different settings and to include progressive and planned measures in order to reach a valuable ECO-SCORE, starting from various baselines. We propose to draw inspiration from the Open Food project^9^, with grades ranging from A to F. Sequential strategies may be used. Balancing factors are often connected to local circumstances and leave room for evolutionary strategies: Table 2.

**Table 2:** ECOSCORE in the bariatric setting.

Main items	Balancing factors
Waste and packaging management	Local obligation in force
Recycling of disposable instruments	Local and inexpensive availability/partnerships
Diminishing/reusing surgical instruments	Voluntary and targeted action or large action, temporary or lasting (ex: anesthesiology)
Recycling anesthetic gas	Technological breakthrough, small or large (ex: endoscopy, robotics)
Energy mix	Social issues, sequential strategies
Other items: water supply, laundry, software and internet, etc	Other actions (ex: corporate green strategy)

Several issues should be addressed in order to avoid misunderstandings:

- Evaluating what can be implemented in a given location at a given time (i.e. ECO-SCORE in a context); examples: particular energy mix, recent initiatives taken at the local level or for instance at the level of a group of hospitals, such as waste policy.
- Addressing the strategic choices that have been or may be included, defined at a regional, national, or multinational level; examples: shift from bariatric surgery to bariatric endoscopy, promotion and/or prohibition of specific procedures. Such choices have an environmental background, but can face controversy at a given time, mostly on scientific grounds: for example, the shift to endoscopy could be implemented if and only if a longer duration of effect can be demonstrated.
- Pondering the potential arbitrations: robotic surgery, Enhanced Recovery After Surgery (ERAS), national or international guidelines, post-operative protocols etc.
- Estimating the factors that are overlooked because they fall into other categories of GHGe. These are typical and explain why figures may not match. A few examples: expenses for transportation, food, etc. for the staff and other employees in hospitals, research, education and training, choice of instruments (disposable or not).

We suggest the items of the ECO-SCORE may be accounted according to Table 3.

**Table 3:** ECO-SCORE marks.

Main items	Balancing factors
Already effective = 4	Already in place = + 1
Implemented immediately = 3	Current impossibility from local issues = − 1
To be implemented soon (< 3 months) = 2	Not ready for use = 0
Impossible to implement for the time being = 1	–

Calculation: A from 20 to 24, B from 16 to 20, etc. + and – accounted for.

### Statements

**Figure 1:**
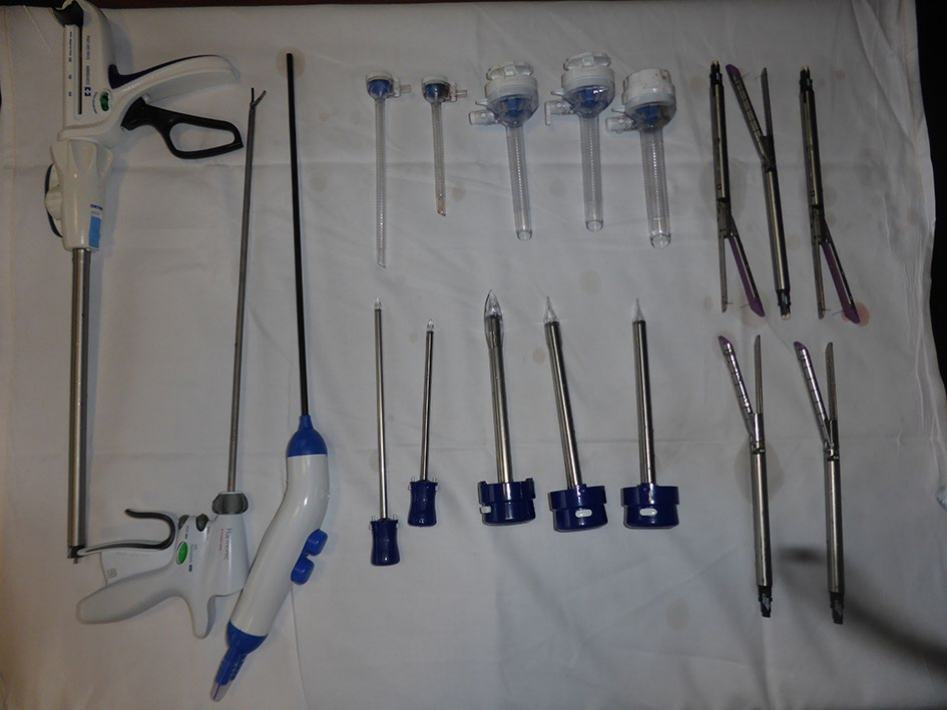
Items to be recycled (disposable instruments), it may be published under a CC BY open access license.

**Figure 2:**
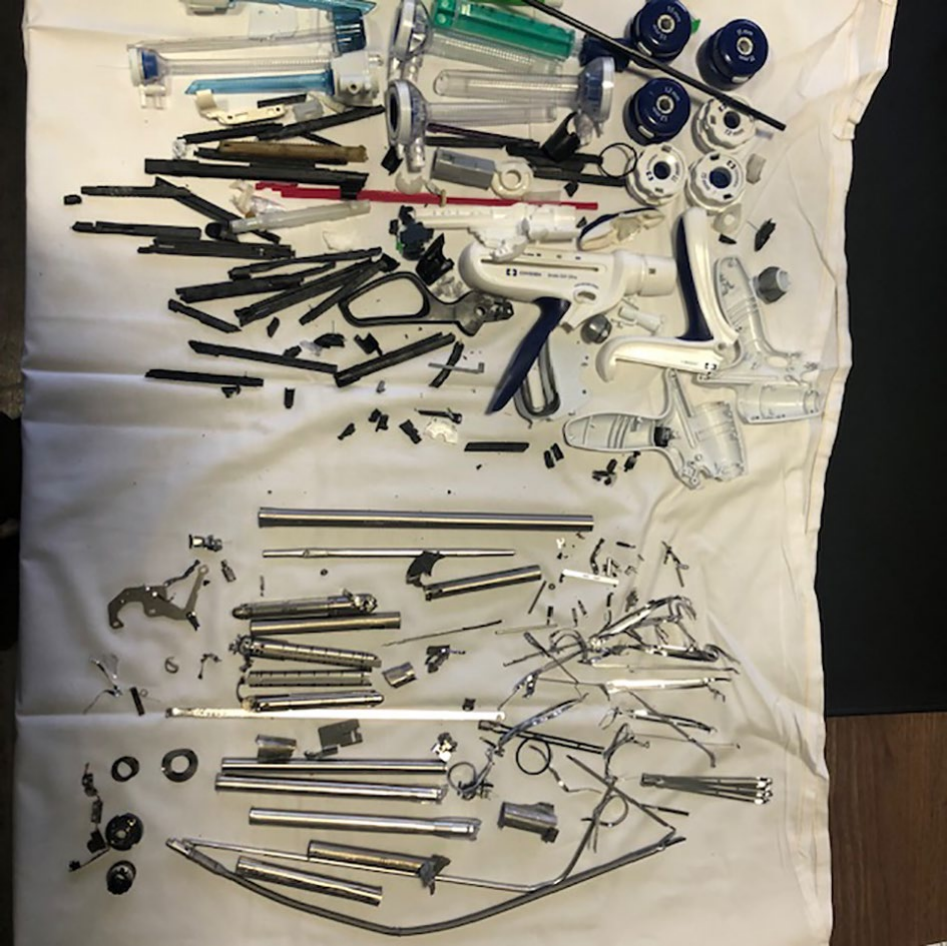
Separation of plastic parts and metallic parts, it may be published under a CC BY open access license.

All procedures were in accordance with the ethical standards of the institutional and/or national research committee and with the 1964 Helsinki declaration and its later amendments or comparable ethical standards. Informed consent was obtained from all individual participants for whom identifying information is included in this article. The study has been pre-approved by the Scientific Committee (Scientific Advisory Board) of the Private Hospitals Units from VIVALTO-France, GCS Merit Vivalto, as a licensing Committee. Figures 1 and 2 have been taken by the author (J Dargent), and therefore need no permission. They may be published under a CC BY open access license; permission is granted to publish them in all format i.e. print and digital.

## Results

### Difficulties encountered to implement the study

- Sparing GHGe is a dynamic and challenging process that currently requires finesse and local negotiation. Not everything is possible in terms of packaging, sterilization process, or reprocessing.
- Recycling surgical instruments was our core initiative (Figs. 1 and 2), since it has been pointed out that there was a high potential in using less surgical tools and/or reusing them^2,3^. Yet, recycling turned out to be tedious, and not cost-efficient: each instrument required 20–40 min to be dismantled, while only 30% of their two components (plastic and metal) could be recycled. As a market item, the recycled material has a highly volatile benefit that is poor for the time being, e.g. at best at the end of 2022 1t Inox = 800–150 € for transportation, other metals = 150€/t, plastic = 150–40 €/t for compacting, not including storage. Moreover, some items contain traces of rare-earth elements that are difficult to recycle. An economic equilibrium is not foreseeable in the next future in this respect, but the initial design of such instruments could be revised in order to facilitate their dismantling, for example in decreasing the glued parts.
- Some of our initiatives were indeed in line with current environmental recommendations, which had not been fully implemented in our hospital: packaging and other waste management, laundry, etc.
- Recycling anesthetic gas canisters represents a recent initiative that has encountered issues of compatibility with current breathing machines. It also conflicted with another recent tool, the AGSS, which conveys anesthetic gases from the OR to the roof to be released in the outside atmosphere.

### Bias

- Global GHGe attributed to the health sector ranges from 6 to 10%; these discrepancies arise from the parameters considered in calculations with on the one hand strictly on-site calculations and, on the other hand, those considering the whole scope of GHGe, e.g. the supply chains, which may account for 59% of these emissions^2^, and the emissions due to staff/patient consumption and transportation, research and development for a given device, etc. Theoretically, only studies using the life cycle assessment (LCA) methodology for all items should be considered for analysis^3^.
- Careful attention should be paid to the boundaries, because otherwise any study would remain speculative, as is the case when dealing with the scope of product inventory for a given functional unit, e.g. a surgical procedure.
- Confusion may arise with regulatory measures that are imposed or advised ahead of common practice. A typical example concerns the anesthesia practice and the gases that are commonly used and/or locally excluded.
- Advocating reusable (e.g. cotton-made) gowns and surgical drapes is relevant, as is reusing/reprocessing instruments, but, depending on each location, it is possible that this measure is encouraged or, on the contrary, that it goes against recommendations regarding infection control.
- The discussion about energy sources covers a variety of situations and energy mixes, for instance in the French Rhône-Alpes area, where this study has been conducted, > 70% of the electricity comes from nuclear energy, which has a very favorable GHGe profile, whereas parts of Germany still rely on coal-electricity supply.
- The example of anesthetic gas emission is compelling: the AGSS has been introduced to protect the personnel from gas emissions inside the ORs, but it also consumes energy and releases GHGe in the atmosphere. The retrieving canister solves this problem, but at an extra-cost that had not been anticipated.
- Advances in surgical techniques can also be challenging, the typical example being robotic surgery. In a study conducted in the field of gynecology by Woods et al., the solid waste generated and energy consumed by robotic surgery represented 40.3 kg CO_2_ eq./patient vs. 29.2 for laparoscopy (+ 38%) and 22.7 for laparotomy (+ 77%)^10^. Returning to laparotomy is barely imaginable, and the robotic upgrade is far from insignificant as it represents an asset in many centers^11^.
- In the study by Thiel^5^ pertaining to laparoscopic hysterectomies, the difference from baseline in GHGe resulting from reprocessing was 9%. The difference resulting from minimizing the instruments was computed at 46%, most of them being seemingly disposable. In our model, we included the recycling of disposable components (staplers and energy devices), which represent the core of some operations (e.g. > 1500 € in a sleeve gastrectomy), and we took into account the emissions resulting from sterilization (autoclave).
- Other limitations: we did not take into account the whole scope of the surgical process, from pre-operative measures to post-operative care, including drug prescription; the computer and email activities have not been included; the water footprint has not been modified; an effort has been made regarding the surgical shirts and trousers for the staff that are no longer disposable, but not regarding the surgical drapes and gowns. Likewise, the duration of the procedures was not relevant, because these are standardized interventions with a limited set of variations (Table 4), the operator and team being the same.

**Table 4:** Operations performed during the two study periods.

	Control group (N = 59)	Action group (N = 56)
Sleeve gastrectomy	38	42
Gastric bypass	6	2
Band removal	14	11
Other	1	1

During the first study period, i.e. the control period, from October 1, 2021 to December 31, 2021, 59 operations were performed, while during the second study period, i.e. the action period, from January 1, 2022 to March 31, 2022, 56 operations were performed (Table 4).

There were neither complications in these two series of interventions, nor intra-operative adverse events that could be attributed to one or the either; the operative time was not different in the two series, regarding the all scope of procedures. None of the measures taken represented an actual “waste of time”.

For 1 laparoscopic sleeve gastrectomy = 12.3 kg CO_2_ eq. saved, for 1 band removal = 5.9 kg CO_2_ eq. saved, and approx. the equivalent for 1 Roux-en-Y gastric bypass, which amounts to 100 kg CO_2_ eq. saved in 3 months, and by extrapolation, 2400 kg in one year (Table 5).

**Table 5:** GHGe saved in a laparoscopic sleeve gastrectomy.

Items (sleeve gastrectomy)	GHGe saved per procedure (kg)	GHGe saved (%)
Autoclave (minimizing instrument use)	0.8	6
Minimizing surgical waste	0.6	20
Sevoflurane reprocessing	4.4 (estimation)	30 (estimation)
Recycling of disposable instruments (staplers and energy devices)	6.4	30
Accessories: reprocessing/reusing, towels, etc	0.1	5
Total (energy not included)	12.3/80 (estimation)	18

### Estimation of our ECO-SCORE

Based on Table 2, we assessed a global SCORE at C+: waste 3+, recycling 3+, diminishing surgical instruments 4−, anesthetic 2, energy 4, other 1.

## Discussion

### Obesity, obesity surgery, and environment

Obesity per se is a major contributor to GHGe, in relation with the carbon footprint of food production and associated supply chain. The genesis of obesity thus significantly impacts GHGe worldwide. Regarding metabolic food waste (MFW), which is defined as the amount of food leading to excess body fat, Europe and North America were found to display the highest values for all three MFW footprints (i.e. carbon, water, and land footprints), being 14 times larger than in South Asia and South-East Asia^12^. The modern food environment, i.e. food availability, also strongly contributes to this genesis in any country^13^.

One would assume that a more or less significant part of this waste may be compensated to a large extent by weight loss, notably achieved by bariatric procedures. While there is no substantial evidence for this, a large body of arguments point to reduced medical costs after surgical weight loss, with therefore a favorable impact on the environment^14^.

Although bariatric procedures marginally contribute to lower resource consumption once patients have achieved sustained weight loss, an argument can be put forward in favor of such interventions, therefore justifying coverage by health insurances: the markedly increased well-being of the obese population^15^. In other words, fighting the stigma that is often associated with obesity is beneficial to this population, since this stigma prevents obese patients from gaining easy access to treatments such as bariatric surgery^16^.

Two current trends may adversely affect this reasoning: (1) the relatively aggressive approach towards Stage I obesity [body mass index (BMI) 30–35], with obesity surgery claiming success in those patients when affected by a comorbidity, typically type 2 diabetes^17^, hence the assertion that metabolic surgery should be strongly promoted and offsets the costs of treating such conditions; (2) the relative extension of robotic surgery in this field also significantly impacts GHGe, as previously shown in gynecological surgery^10^ or other types of surgery^11^.

### Looking for opportunities and compromises

Other strategies pursuing common goals are currently being considered. On the one hand, “green surgical innovation”^18^ suggests that evaluating new surgical devices or new surgical options in general, such as robotics^10,11^ or digitalized options, could benefit from the strict analysis of their carbon footprint, which thus determines whether a new strategy/device should be implemented or not. This is questionable, since innovations and environment may remain compatible. On the other hand, others rightly point out that surgical issues have boundaries that go beyond the strict perimeter of the OR^19^, or even suggest a much broader move that encompasses several items in order to build sustainable and resilient surgical systems, possibly at the level of a geographical area, e.g. the West Pacific region, including infrastructure, service delivery, finance, information systems, health workforce, and governance^20^. One may object that health systems are closely interconnected throughout the world, for example when it comes to the workforce, and that such definitions may be vague enough to hamper real and coordinated efforts.

As noted by Rizan et al.^3^, the numbers are difficult to interpret because the various studies have different frameworks and use different references, with the authors placing emphasis on the various choices that can be debated, e.g. whether or not including anesthesiology, energy mix, life cycle analysis, etc. Hence, we suggest acting upon what is currently within reach at a given time and evaluating the progress that can be achieved at various levels. We propose to start from a given situation in a hospital and try to improve different scores, thereby contributing to a more global effort including recycling, re-processing, eliminating single-use items whenever possible, energy saving, and minimizing instrument use and anesthetic gas. It is common to feel that others should make environmental efforts before we do, or in other words, that other fields have a more detrimental impact on the environment than our own. In view of the substantial contribution of the Scope 2 (energy) to GHGe as compared to the others, one may claim to be powerless or favor green-washing options and focus on good intentions rather than real actions. Likewise, many choose to blend environmental issues with social issues when presenting results, which is politically relevant (at least regarding the so-called “social and environmental responsibility”), but probably scientifically irrelevant in the medical field.

Yet, it is interesting to look at other fields whose environmental strategy is nowadays being questioned, such as the automobile industry, fashion industry, construction, computer software and internet, etc. How fast are efforts being made and should we follow the same path? What kind of pressure is applied in each case? These are difficult questions, and it may be best that everyone acts on their own behalf, regardless of what others do.

For now, we overlooked the final aspect of GHGe in patients at the very end of the surgical process, i.e. considering the economical long-term benefits of weight loss, which offset the initial costs in many studies. For instance, the decrease in drug costs has been evaluated: In a meta-analysis performed by Lopes et al. in 2015, the mean reduction in total drug costs was estimated at 49.8% over a follow-up duration of 6–72 months after bariatric surgery^21^. Yet, such studies are lacking for GHGe, and we need benchmarks. One study has been conducted in the field of esophageal reflux surgery, showing that the cost–benefit ratio was not favorable up to 9 years after surgery^22^. Further studies are warranted to assess the benefits of GHGe reduction in the bariatric field.

Lastly, we addressed what could be called the “obesity debt”, i.e. the food waste associated with overweight and obesity, which amounts to 140 M tons/year according to Totti et al.^12^. This does not impede the efforts towards weight loss, on the contrary, but it could be an incentive to favor less energy-consuming methods, e.g. endoscopic solutions rather than typical laparoscopic surgical options^23^. While an ultimatum like the EU ban of combustion-engine cars by 2035 is barely conceivable, it makes sense to promote incentives to develop less impacting technologies such as endoscopic bariatric methods, perhaps associated with new drugs (GLP-1 receptor agonists).

There is no reason why bariatric surgery would be more or less environmentally friendly according to BMI range; yet adding more patients to the surgical workflow seems unfriendly to the same environment, particularly if alternative and sound options exist for those patients (drugs, endoscopy, etc.), which is the case for lower BMI patients, and even for selected higher BMI patients and/or unwilling to undergo surgery. To put it differently: such treatments, that are explicitly non-surgical, entail less GHGe, and are therefore more likely to concern this range of BMI (30–35). However, when surgery is performed in these patients, it does not mean more GHGe, but those could have been spared if surgery had not been the primary option.

## Conclusion

Is it cost-effective to try and diminish GHGe (and other items) in an operative setting? Does it affect surgical outcomes? Basically, the efforts we can make without further delay are mostly cheap or affordable, more strategic ones (e.g. shifting to less consuming operations) represent a different issue that would require funding/incentives and consensus. The efforts that we tried are allegedly quite doable even if facing reluctance; they did not and they should not impact the medical/surgical outcomes at all.

## Data Availability

Data will be made available on reasonable request; contact: Jerome Dargent, jerome.dargent@polyclinique-rillieux.fr.
